# Preparation of a Novel Perilla Essential Oil/Grape Seed Extract–Chitosan/Gelatin Composite Edible Gel Film and Its Application in the Preservation of Grass Carp

**DOI:** 10.3390/gels11050321

**Published:** 2025-04-25

**Authors:** Shan Xue, Rui Xu, Jia Liu

**Affiliations:** 1 College of Biological Science and Technology, Minnan Normal University, Zhangzhou 363000, China; 2 Research Institute of Zhangzhou-Taiwan Leisure Food and Tea Beverage, Zhangzhou 363000, China; 3 Zhangzhou Food Science Research Institute, Zhangzhou 363000, China; 4 Guizhou Academy of Agricultural Sciences, Guiyang 550006, China

**Keywords:** composite gel films, perilla essential oil (PE), grape seed extract (GSE), preparation, fish preservation

## Abstract

In this study, a new edible gel of Perilla essential oil (PE)/grape seed extract (GSE)–chitosan/gelatin was prepared, and it was applied to the preservation of silver carp. By establishing a fuzzy mathematical model, using a single-factor experiment and Box–Behnken response surface optimization combined with matlab analysis, the optimum preparation conditions of composite gel films were determined: the addition of PE (*p* < 0.01) was 6.91 μL/mL, the addition of GSE (*p* < 0.05) was 0.45 mg/mL, and the addition of gelatin (*p* > 0.05) was 1.63%. Under these conditions, the composite gel films exhibited an excellent water vapor barrier and mechanical properties. Using Fourier-transform infrared spectroscopy (FTIR) analysis, it was found that the addition of PE enhanced or weakened the absorption peaks, indicating the molecular interaction between PE and the substrate. Scanning electron microscopy (SEM) observed that the surfaces of the composite gel films with added PE were smooth, but there were a few pores in the cross-section. X-ray diffraction (XRD) analysis showed that PE had good compatibility with other components. The fresh-keeping experiment showed that the composite gel films could significantly prolong the fresh-keeping period of grass carp. After 10 days of storage at 4 °C, compared with the blank group (without plastic wrap) and the control group (with composite gel film, no PE added), the experimental group (with composite gel films, PE added) showed better fresh-keeping effect in terms of sensory score, moisture content, pH value, TBARS value, and TVB-N value (*p* < 0.05). Correlation analysis further confirmed the positive effects of composite gel films on water content, pH value, TVB-N, and other quality indexes of silver carp, indicating that the composite gel films will have broad application prospects in the food preservation field. This study provides an innovative basis and theoretical basis for the development and application of natural polysaccharide/protein composite edible film, which is helpful to promote the development of green food-packaging materials.

## 1. Introduction

As natural polymer materials, gelatin and chitosan have attracted much attention in the field of food preservation due to their degradability, biocompatibility, and film forming properties. Gelatin is obtained by the hydrolysis of collagen and amino acid residues such as glycine, and proline in its molecular chain can form thermoreversible gels through hydrogen bonding, giving the film an excellent oxygen barrier property and flexibility [1]. However, the single adhesive film has defects such as low mechanical strength and poor moisture resistance. Chitosan, as the deacetylation product of chitin, can protonate the amino group on its molecular chain to form cationic properties, showing significant inhibition on common foodborne pathogens such as *Escherichia coli* and *Staphylococcus aureus*, and can improve the mechanical properties of the film through intermolecular hydrogen bonding [2]. The application of composite membrane solves the application limitations of a pure chitosan membrane, such as high brittleness and poor light transmittance [3].

In recent years, the complex system of gelatin and chitosan has become a research hotspot. The three-dimensional network structure formed by electrostatic interaction and hydrogen bond cross-linking can significantly improve the film’s performance [4,5]. In order to further expand its function, the current research mainly introduced functional components through blending modification technology. Studies suggest that plant essential oil has high antibacterial and antioxidant activities, which can reduce or replace synthetic food additives [6]. When essential oil was added to edible films, it was applied to meat and meat products for storage, and the preservative and preservation effect was significantly improved [7]. Perilla essential oil (PE) is derived from the leaves or shoots of Perilla plants of the Lamiaceae family and is often extracted by steam distillation. Its main components include perillaldehyde, perillone, limonene, eugenol, terpene, etc. It has antibacterial, anti-inflammatory, antioxidant, skin soothing, blood circulation promotion, and other effects and has significant antibacterial and fresh-keeping functions in the application of food plastic film [8]. In addition, studies have shown that the use of nanoemulsion technology can improve the dispersion and stability of PE in the mixing system and extend the shelf life of food [9].

At the same time, grape seed extract (GSE), as a natural active ingredient, mainly comes from the seeds of grape fruits, and its main components include procyanidins, catechins, epicatechins, gallic acid, and other polyphenols. These ingredients have powerful antioxidant capabilities and are among the most potent antioxidants currently known from plant sources [10]. This study showed that GSE can significantly improve the antioxidant properties of edible film and delay the deterioration of food oxidation. For example, adding it to chitosan or soy protein composite membranes can improve the mechanical properties and barrier properties of the membranes, while giving the membranes antibacterial and antioxidant functions [11]. In addition, GSE can continue to play a fresh-keeping role and extend the shelf life of food through the slow-release mechanism [12]. The application of this natural extract not only improves the functionality of the edible film but also conforms to the developing trend of green packaging.

Based on the above analysis, this study prepared a new type of PE/GSE–gelatin/chitosan composite edible gel film. By establishing a fuzzy mathematical model, using a single-factor experiment and Box–Behnken response surface optimization combined with matlab analysis, the preparation process of the gel films was optimized, the characteristics were characterized, and it was applied to the fish preservation of grass carp in order to provide an innovative basis and a theoretical basis for the development and application of natural functional polysaccharide/protein composite edible gel films.

## 2. Results and Discussion

### 2.1. Results of Single Factor

#### 2.1.1. Effect of GSE Addition on Properties of PE/GSE–Gelatin/Chitosan Composite Edible Gel Films

**Figure 1:**
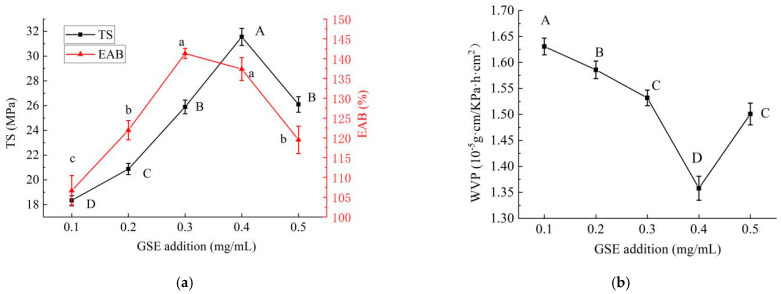
Effects of GSE addition on properties of PE/GSE−gelatin/chitosan composite edible gel films ((**a**) Mechanical properties (tensile strength (TS) and elongation at break (EAB)), (**b**) Water Vapor Permeability (WVP)). (The different letters A–D marked in the figure (**a**) indicated significant differences in the results of TS (*p* < 0.05); The different letters a–c marked in the figure (**a**) indicated significant difference in EAB (*p* < 0.05); The different letters A–D marked in the figure (**b**) indicated significant differences in the results of WVP (*p* < 0.05).).

The effects of different amounts of GSE on the mechanical properties of edible composite gel films are shown in Figure 1a. The results showed that with the increased GSE addition, the TS value and the EAB value of composite gel films first increased and then decreased (*p* < 0.05); the TS value reached the maximum value when the GSE supplemental level was 0.4 mg/mL, and the EAB value reached its peak when the GSE supplemental level was 0.3 mg/mL. This may be due to the interaction of phenolic hydroxyl groups in GSE with hydrogen bonds in chitosan to increase the toughness of the membrane. However, the excessive addition of GSE will reduce the cross-linking effect between chitosan and fish gelatin, which will reduce the toughness of the membrane and lead to a decrease in the maximum elongation at break of the composite membrane [13].

The effects of different amounts of GSE on the WVP of the gel films are shown in Figure 1b. With an increase in GSE, the WVP showed a trend of first overall decreasing and then increasing (*p* < 0.05). When the GSE content was 0.4 mg/mL, the WVP had the smallest value. This may be because the polyphenols (such as proanthocyanidins) in GSE can cross-link with gel materials (such as proteins and polysaccharides) to enhance the intermolecular force and form a denser network structure, thus reducing the water vapor diffusion channel [14]. At the same time, GSE, as a filler, may block micropores or cracks in the membrane and hinder the migration of water molecules. However, an excessive addition may lead to phase separation or aggregation, damage the uniformity of the membrane, and form more water vapor diffusion paths. Moreover, the introduction of hydrophilic groups (such as hydroxyl groups) may increase the hygroscopicity of the membrane and promote the penetration of water molecules, so the WVP increases [15]. After comprehensive consideration, the GSE dosage of 0.4 mg/mL was selected for the follow-up test.

#### 2.1.2. Effects of PE Addition on Properties of PE/GSE–Gelatin/Chitosan Composite Edible Gel Films

**Figure 2:**
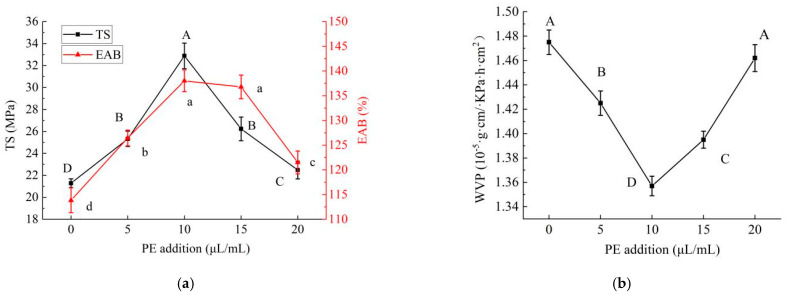
Effects of PE addition on properties of PE/GSE−gelatin/chitosan composite edible gel films ((**a**) mechanical properties (tensile strength (TS) and elongation at break (EAB)), (**b**) Water Vapor Permeability (WVP)). (The different letters A–D marked in the figure (**a**) indicated significant differences in the results of TS (*p* < 0.05); The different letters a–d marked in the figure (**a**) indicated significant difference in EAB (*p* < 0.05); The different letters A–D marked in the figure (**b**) indicated significant differences in the results of WVP (*p* < 0.05).).

The effects of the PE addition on the properties of the gel films are shown in Figure 2a. It can be seen from the figure that with the increase in the addition of PE, the TS value first increases and then decreases (*p* < 0.05), indicating that the strength or hardness of the material reaches the maximum at 10 μL/mL. The EAB value also increased first and then decreased with the increased PE addition, and the highest value appeared at 10 μL/mL. There was no significant difference in the EAB value when the PE addition was 10 μL/mL and 15 μL/mL, indicating that the absorption rate or expansion rate of the gel films was the largest at this time. The trends of the two indicators were roughly similar, and the amount of PE added when the indicators reached the peak was the same (both 10 μL/mL).

The effects of different amounts of PE on the mechanical properties of the gel films are shown in Figure 2b. The WVP value first decreased and then increased with the increase in the PE supplemental level (*p* < 0.05). This indicated that the addition of PE can reduce the WVP in the samples in the range of 0–10 μL/mL. At 10 μL/mL, the WVP value was the lowest, which indicated that the barrier property of the material was the best. This may be because at this addition, PE interacted most effectively with the material matrix, forming a tighter structure that reduced water vapor transmission. However, after more than 10 μL/mL, continuing to increase the addition of PE will increase the WVP.

The reason for this phenomenon may be due to the good interaction between the active ingredients in PE (such as Perilla aldehyde, limonene, etc.) and the substrate materials such as chitosan and gelatin. These components can form a tighter network structure with polysaccharides and proteins through hydrogen bonding or hydrophobic interactions, thereby improving the mechanical properties of the film and effectively reducing the water vapor transmission channel, thereby reducing the WVP value [16]. At the same time, a suitable amount of PE can increase the flexibility of the gel films, so that it can better disperse the stress during the stretching process, so as to improve the EAB, and the right amount of PE can fill the micropores or cracks in the film, further enhance the barrier property of the film, and reduce the WVP. However, when the amount of PE added exceeds a certain threshold, it may lead to phase separation. Too much PE addition was not evenly dispersed in the matrix, forming independent phases or aggregation areas, which have poor mechanical properties, thereby reducing the strength of the overall film. In addition, PE may form nanoemulsions with macromolecular chitosan and gelatin. The synergistic effect of nanoparticles and dynamic cross-linking enabled the gel films to possess both high strength and high toughness, breaking through the performance bottleneck of traditional polysaccharide protein materials [17]. The addition of nanofibers constructs a multi-scale cross-linked network, which enhances the rigidity of the gel films while maintaining a high elongation at break through the mechanisms of sacrificial bond breakage and molecular chain rearrangement [18]. However, when the PE exceeds a certain amount, it may interfere with the cross-linking between chitosan and gelatin, disrupt the original network structure, and lead to a decline in the mechanical properties and water vapor barrier performance of the gel films [19].

#### 2.1.3. Effects of Gelatin Addition on Properties of PE/GSE–Gelatin/Chitosan Composite Edible Gel Films

**Figure 3:**
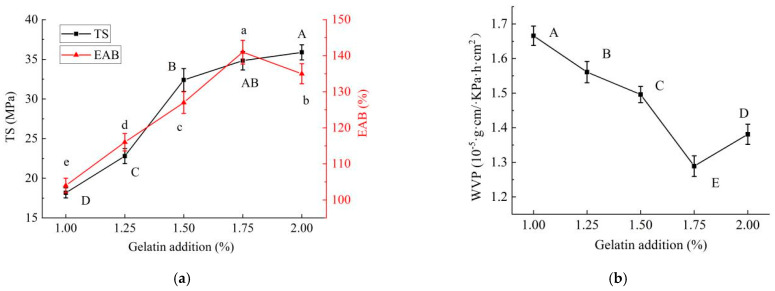
Effects of gelatin addition on properties of PE/GSE−gelatin/chitosan composite edible gel films ((**a**) mechanical properties (tensile strength (TS) and elongation at break (EAB)), (**b**) Water Vapor Permeability (WVP)). (The different letters A–D marked in the figure (**a**) indicated significant differences in the results of TS (*p* < 0.05); The different letters a–e marked in the figure (**a**) indicated significant difference in EAB (*p* < 0.05); The different letters A–E marked in the figure (**b**) indicated significant differences in the results of WVP (*p* < 0.05).).

The effects of different gelatin dosages on the mechanical properties of the composite edible gel films are shown in Figure 3. The results show that with the increase in gelatin content, the TS of the composite films increased significantly (*p* < 0.05), and the EAB gradually increased and then decreased (*p* < 0.05) (Figure 3a). This may be because, when the gelatin content is in the range of 1.0–2.0%, a large number of the amino (-NH_2_) and carboxylic (-COOH) groups in the gelatin molecular chain can form a more dense three-dimensional network structure with polysaccharide chitosan through hydrogen bonding, electrostatic interaction, or covalent cross-linking, improving the continuity of the gel film and thereby improving the mechanical strength [20]. At the same time, when the gelatin content is in the range of 1.0–1.75%, a large number of flexible regions in the gelatin molecular chain (such as the helical structure composed of proline and hydroxyproline) can give the films good ductility, and the molecular chain slips or rearranges during stretching, delaying the fracture. However, when the amount of gelatin added continues to increase, excessive gelatin may lead to phase separation, forming interface defects and rapid crack expansion during stretching, resulting in a significant decrease in EAB [20].

As can be seen from Figure 3b, with the increase in the gelatin supplemental level, the WVP value of the composite gel films showed a trend of significant decrease and then increase (*p* < 0.05). When the gelatin content was 1.75%, the WVP value was the lowest, indicating that the water resistance of the gel film was the best. This may be because at this addition, the gelatin interacts most effectively with the material matrix, forming a tighter structure and thus reducing water vapor transmission [21], which was consistent with the 3(a) result.

#### 2.1.4. Effect of Glycerol Addition on Properties of PE/GSE–Gelatin/Chitosan Composite Edible Gel Films

**Figure 4:**
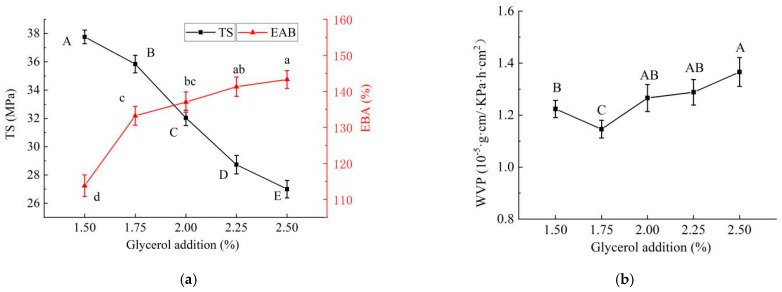
Effects of glycerol addition on properties of PE/GSE−gelatin/chitosan composite edible gel films ((**a**) mechanical properties (tensile strength (TS) and elongation at break (EAB)), (**b**) Water Vapor Permeability (WVP)). (The different letters A–E marked in the figure (**a**) indicated significant differences in the results of TS (*p* < 0.05); The different letters a–d marked in the figure (**a**) indicated significant difference in EAB (*p* < 0.05); The different letters A–C marked in the figure (**b**) indicated significant differences in the results of WVP (*p* < 0.05).).

The effects of different glycerol addition amounts on the mechanical properties of edible composite films were shown in Figure 4a. As shown in Figure, the TS of the composite gel films gradually decreased with the increase in the addition of glycerol (*p* < 0.05). Glycerol is a hydrophilic small-molecule polyol, which can be inserted between the molecular chains of film-forming materials (such as proteins and polysaccharides), weaken the intermolecular hydrogen bonds and van der Waals forces, and increase the mobility of the molecular chains. As a result, the film becomes softer and more flexible, which may result in a gradual decrease in the TS of the gel films [19]. An appropriate amount of glycerol (low concentration) may improve the fluidity of the film-forming solution and reduce the micropores or cracks formed during the drying process, thus maintaining a certain tensile strength, but an excessive addition will destroy the orderly arrangement of molecular chains and lead to a loose structure [22]. Glycerol, as a high efficiency plasticizer, can significantly increase the break elongation of edible film, especially for polysaccharide-based or brittle matrices. However, as the amount of glycerol added continues to increase, the plasticizing effect of glycerol is limited, and the improvement of EAB is also limited [22].

As can be seen from Figure 4b, with the increase in the glycerol supplemental level, the WVP of the gel film showed a trend of first decreasing and then increasing (*p* < 0.05), and when the glycerol supplemental level was 1.75%, the WVP had a minimum value. This may be because a small amount of glycerol (<5%) at a low concentration addition may fill micropores or cracks in the film, reduce defects, and temporarily reduce the WVP. However, with the increase in the concentration, the plasticizing effect of glycerol gradually dominates. Glycerol is a strong hydrophilic small molecule, and its hydroxyl group (OH) can form hydrogen bonds with water molecules, significantly improving the hygroscopic property of the film, promoting the adsorption–diffusion process of water vapor in the membrane, resulting in an increase in WVP [23]. Therefore, taking comprehensive consideration, the additional amount of glycerol was selected as 1.75%.

### 2.2. Results of Box–Behnken Design and Matlab Analysis

#### 2.2.1. Model Building

In order to obtain the optimal preparation process conditions of the composite gel films, and make them have good mechanical properties and a low WVP at the same time, the values of TS, EAB, and WVP were used as response values for regression analysis, according to the Box–Behnken design. Three factors, PE addition (A), GSE addition (B), and gelatin addition (C), were used as independent variables, and the experimental design and results are shown in Table 1. The membership degree values of fuzzy comprehensive evaluation indexes and the results of fuzzy comprehensive evaluation are shown in Table 2.

#### 2.2.2. The Results of Response Surface Analysis and the Significance Test

According to the test results in Table 2, Design Expert 8.0.6.1 software was used to analyze the test data, and the response surface regression simulation equation of the fuzzy comprehensive evaluation (Y) was obtained as follows (Formula (1)):

(1) $$Y=0.7114-0.0827\times A-0.0201\times B+0.0166\times C+0.0335\times AB-0.0335\times AC-0.0447\times BC-0.0923\times A^{2}-0.0631\times B^{2}-0.0651\times C^{2}$$

The validity of the above regression equation was tested based on the effects of A, B, and C on the Y value, which are shown in Table 2 (see Table 3). As can be seen from the table, the F-value is an important index to evaluate the impact of each variable on the response surface. The larger the F-value, the greater the impact of the variable on the response. The Y (fuzzy mathematical comprehensive score) model was extremely significant (*p* < 0.01), and the item of lack of fit was not significant (*p* > 0.05), indicating that the interference factor of the equation was small, and the fitting effect of the independent variable and the dependent variable was good. The regression coefficient R^2^ was 0.9782, the regression equation correction determination coefficient R_Adj_^2^ = 0.9502, the AP value = 15.056, and the coefficient of variation (CV) = 3.66%. The results showed that the regression equation had high reliability and good reproducibility and reliability. In conclusion, it was reasonable to establish a model based on the mechanical properties and WVP of the gel films as the response values. In terms of influence degree, A (PE) > B (GSE) > C (gelatin). In the Y model, the interaction of AB and AC pairwise factors was significant (*p* < 0.05), and the interaction of AC factors was extremely significant (*p* < 0.01). When the addition of PE was 6.91 μL/mL, the addition of GSE was 0.45 mg/mL, and the addition of gelatin was 1.63%, the fuzzy mathematical comprehensive score of PE/GSE–gelatin/chitosan composite edible gel films had the maximum value (0.7456). This model can be used to analyze and predict the optimum process parameters of composite gel film preparation.

#### 2.2.3. Response Surface Interaction and Optimization Results Analysis

**Figure 5:**
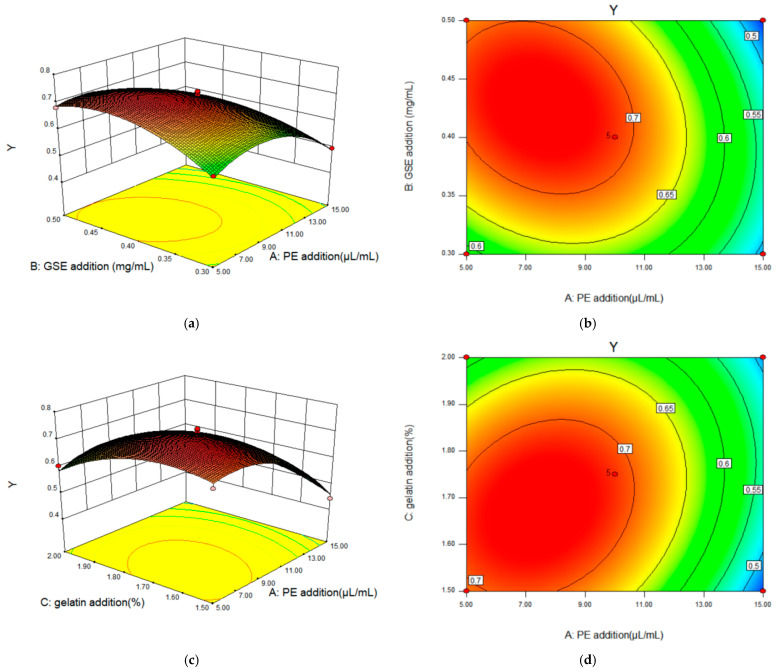

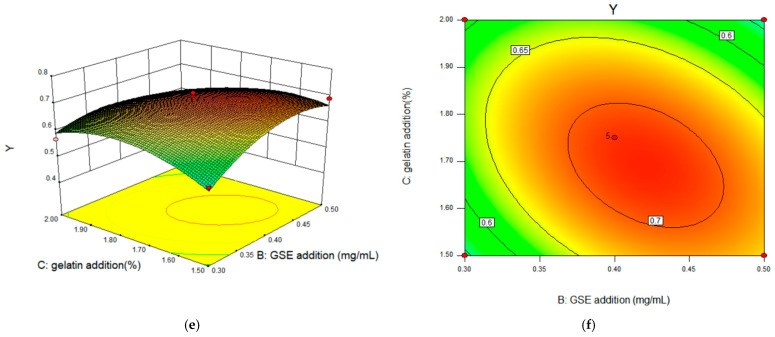
Contour and response surface diagram of interaction between PE addition (A), GSE addition (B), and gelatin addition (C) on Y (fuzzy mathematical comprehensive score). ((**a**,**c**,**e**) represented the response surface plots of the interaction of AB, AC and BC, respectively. (**b**,**d**,**f**) represented the contour plots of the interaction of AB, AC and BC, respectively.).

Through the regression model analysis, the interaction between the influencing factors can be obtained (see Figure 5). Figure 5a–f, respectively, show the pair-to-pair interaction of the other two factors on the response surface index under the condition that one factor was fixed. The strength of the interaction effect between the two variables can be better reflected by the shape of the contour map. The ellipse of the contour line shows that the interaction between the two factors was significant, while the circle does not. In addition, the response surface 3D graph can intuitively reflect the impact of various factors on the response value.

According to Figure 5a,c,e, it can be seen that the contours of the interaction terms PE addition (A) and GSE addition (B) and PE addition (A) and gelatin addition (C) are all ellipses, and the solid map of the response surface is very steep. Therefore, the interaction between A and B and A and C was strong, and the influence on the comprehensive score of fuzzy mathematics was significant. The ellipses of the contours of the interaction terms GSE addition (B) and gelatin addition (C) are more pronounced, and the stereogram of the response surface is steeper, so the interaction between B and C is more significant than AB and AC, which is the same as the results of the ANOVA. As can be seen from the contour lines in Figure 5b,d,f, there is a maximum value of the fuzzy mathematics comprehensive score under each interaction, which indicates that each factor has a corresponding optimal value.

#### 2.2.4. Matlab Analysis

Matlab (MATrix LABoratory) software (R2021a) is one of the best scientific and technological application softwares in the world. It has powerful scientific calculation functions and provides a special optimization toolbox. It is widely used in various research fields by establishing mathematical models of research problems and writing program codes to effectively calculate optimal solutions [24,25]. Matlab can not only obtain the 4D interaction surface based on Y optimization through programming but also obtain the optimal process value range corresponding to the value of a certain influence factor (different high, medium, and low) and further calculate the interactive influence among factors [26].

**Figure 6:**
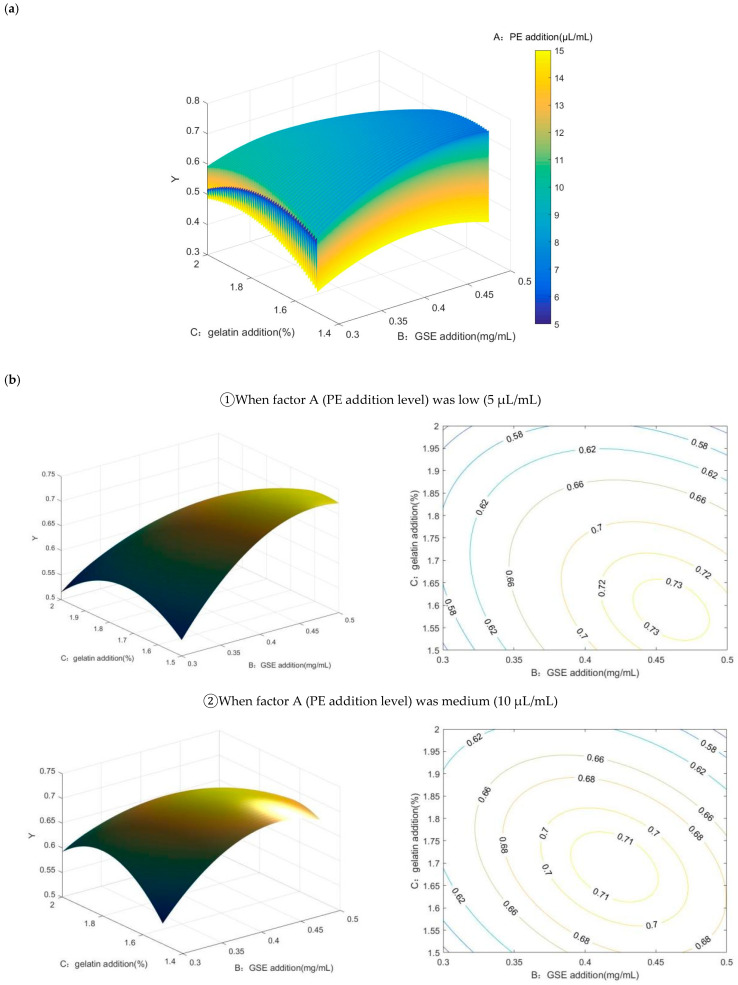

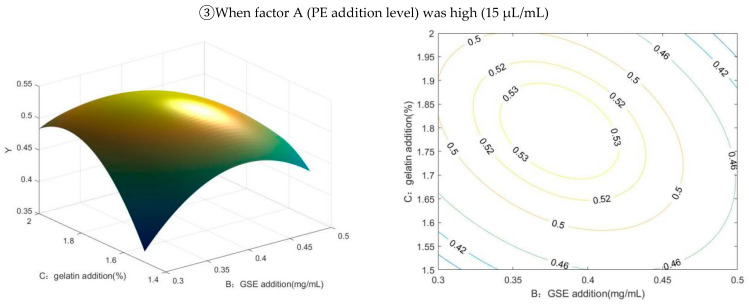
Four-dimensional and three-dimensional interactions among factors optimized based on Y ((**a**) the 4D interactive surface based on the optimization of Y; (**b**) 3D contour plots and response surface plots of the effects of the interaction of various factors on Y). (In the (**b**), When factor A (PE addition level) was low (5 μL/mL), medium (10 μL/mL), high (15 μL/mL), the 3D contour plots and response surface plots of the effects of the interaction of BC on Y were shown, respectively.).

Since the previous results showed that factor A (PE addition) had the most significant effect on the gel films, in order to better describe the interactive effects among the analysis data, we plotted the interactive effects of gelatin addition and GSE addition amounts on Y in 4D, when factor A (PE addition level) was low (5 μL/mL), medium (10 μL/mL), and high (15 μL/mL), respectively. The three dimensions of rotary surface and contour projection were plotted as shown in Figure 6.

When the addition of PE, (A) was set at a low value (A = 5 μL/mL), the supplemental level of GSE (B) was fixed, and with the increase in the supplemental level of gelatin (C), the Y value showed a slight increase at first, and then a significant trend of continuous decline. The value of Y ranged from 0.5147 to 0.7341. When B was 0.43–0.48 mg/mL and C was 1.5–1.65%, Y could approach a larger value.

When the addition of PE (A) was the middle value (A = 10 μL/mL), the additive amount of GSE (B) was fixed, and the Y value first increased and then decreased with the increase in the additive amount of gelatin (C). In this case, the Y value ranged from 0.5350 to 0.7154. When B was 0.38–0.45 mg/mL and C was 1.6–1.75%, Y could approach a larger value.

When the supplemental level of PE (A) was higher (A = 15 μL/mL), the supplemental level of GSE (B) was fixed. With the increase in the supplemental level of gelatin (C), the Y value first increased and then decreased, which was similar to the change trend when A = 10 μL/mL. The value of Y ranged from 0.3599 to 0.5391. When B is 0.325–0.425 mg/mL and C is 1.65–1.9%, Y can approach a larger value.

In summary, when the addition of PE (A) is at a lower level (A = 5 μL/mL), a larger theoretical value of Y can be obtained. At this time, when B is 0.43–0.48 mg/mL and C is 1.5–1.65%, Y can obtain a larger value.

#### 2.2.5. Verification Test

**Figure 7:**
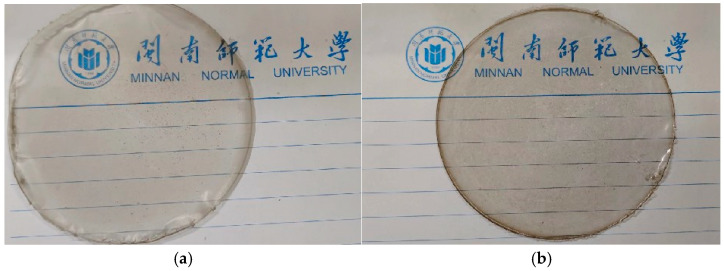
Picture of PE/GSE–gelatin/chitosan composite edible gel films. ((**a**) GSE–gelatin/chitosan composite edible gel films (no PE) (CG); (**b**) PE/GSE–gelatin/chitosan composite edible gel films) (EG).

According to the analysis results of Design-Expert 8.0.6.1 software, the optimal preparation conditions of composite gel films were obtained as follows: the additional amount of PE was 6.91 μL/mL, the additional amount of GSE was 0.45 mg/mL, and the additional amount of gelatin was 1.63%. Under these conditions, the fuzzy mathematical score of the gel films was 0.7456. In order to verify the reliability of the extraction condition, three parallel tests were carried out under this condition, and the results showed that TS was 23.81 ± 0.22 MPa, EAB was 172.53% ± 3.05, and WVP was 1.179 ± 0.07 (10^−5^ g cm/(KPa h kPa·cm^2^). The fuzzy mathematical score of the gel film was 0.7581 ± 0.0119, which is close to the theoretically predicted value, and the relative error between the actual value and the predicted model was about 1.6765%, indicating that the method can be used to analyze and predict the best process for the composite film. The macro images of the gel films are shown in Figure 7. The control group (CG) was GSE–gelatin/chitosan composite edible gel films (without PE added), and the experimental group (EG) was PE/GSE–gelatin/chitosan composite edible gel films with 10 μL/mL of PE added.

### 2.3. Results of Fourier-Transform Infrared Spectrometer (FTIR)

**Figure 8:**
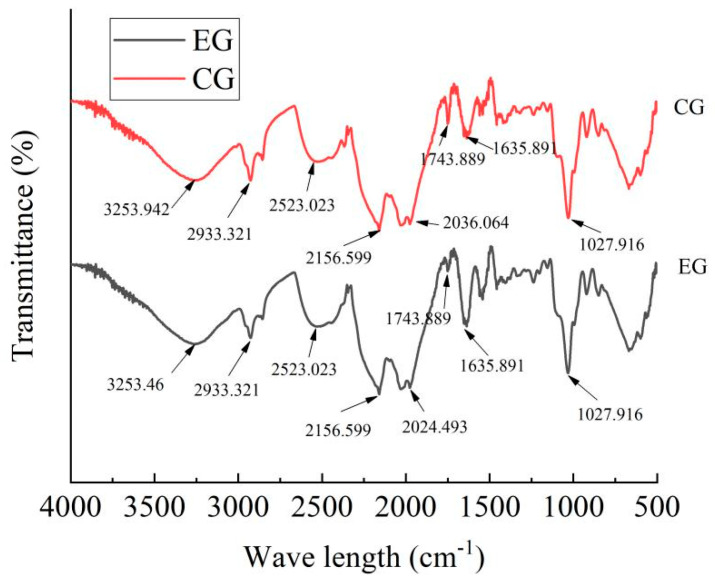
FTIR analysis of the edible gel films (CG: GSE–gelatin/chitosan composite edible gel films (no PE); EG: PE/GSE–gelatin/chitosan composite edible gel films).

The FTIR analysis of the gel films of the experimental group (EG, PE/GSE–gelatin/chitosan composite edible gel films with 10 μL/mL of PE added) is shown in Figure 8. The GSE–gelatin/chitosan composite edible gel films without PE added were taken as the control group (CG) to observe the effect of the PE addition on the gel film’s structure. According to the analysis of infrared spectra, the spectra of the above two films were highly similar, and the characteristic peaks were mainly in the range of 3180~3375 cm^−1^ (amido-A band, N-H stretching vibration, and O-H stretching vibration), 2900 cm^−1^ (asymmetric stretching vibration of CH_2_ and CH_3_), 1650–1600 cm^−1^ (amylamine I band, C=O stretching vibration), 1550–1500 cm^−1^ (amylamine II band, N-H bending vibration), and 1240 cm^−1^ (stretching vibration of amide III band, C-N bond) [27]. Among them, the absorption peaks of the composite films supplemented with PE mainly increased or decreased at 1635–1743 cm^−1^, which may be due to the C=O stretching vibration changes in aldehydes or lipid carbonyl groups in PE [13]. At the same time, the very small absorption peaks between 1600–1500 cm^−1^, 1450–1400 cm^−1^, and 1300–1200 cm^−1^ were also changed, which may be due to the fact that certain components of the essential oil (such as limonene, β-clove hydrocarbon, etc.) may be enhanced or weakened in a certain wavenumber range.

Furthermore, the blue shift in the O-H peak (3253.942 cm^−1^) confirmed the strengthening of the hydrogen bonds or the formation of new hydrogen bonds. The enhancement of hydrogen bonds and ester group cross-linking significantly improved the tensile strength of the material. Hydrogen bonds, as physical cross-linking points, increased the interaction forces between molecular chains, enabling the material to disperse stress more effectively during stretching and thereby enhancing the tensile strength [28,29]. However, this change may also lead to the elongation at break not reaching the optimal value. This change makes the material structure more compact and reduces the free volume. This densification will prolong the diffusion path of water vapor molecules, thereby reducing the water vapor transmission rate. This is consistent with the results of the single-factor experiment in Section 2.1. This was why it was necessary to establish a comprehensive index to balance TS, EAB, and WVP, so that the gel films had both good mechanical properties and water vapor barrier properties simultaneously.

### 2.4. Results of Scanning Electron Microscope (SEM)

**Figure 9:**
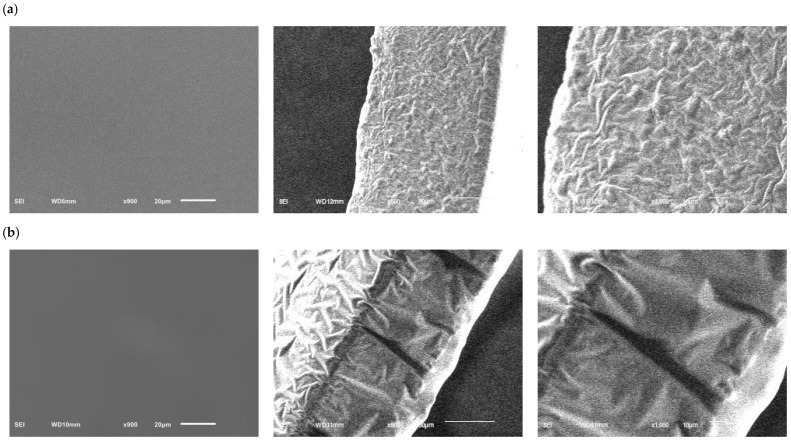
Microstructure of the composite edible gel films (**a**) surface images (magnification: 900× *g*), cross-sectional images (magnification: 500× *g*), and cross-sectional images (magnification: 1000× *g*) of CG; (**b**) surface images (magnification: 900× *g*), cross-sectional images (magnification: 500× *g*), and cross-sectional images (magnification: 1000× *g*) of EG, respectively.

The surface and cross-section SEM images of the gel films of the CG and EG are shown in Figure 9. By observing the surface morphology of the composite gel films, it can be seen that the surfaces of the CG and EG were both smooth, without obvious pores and wrinkles, and no obvious differences are seen. By observing the cross-sectional morphology of the composite film, it can be seen that both the CG and EG presented a dense network structure, with high density and good intermolecular cross-linking. The difference was that the composite gel films with added PE showed some pores and grooves. The reasons for this phenomenon may stem from phase separation, solvent evaporation, and changes in the cross-linking network [30,31]. The phase separation of PE as a hydrophobic lipid from the polysaccharide matrix significantly increased the porosity of the gel films and explained the influence of interfacial separation on the microstructure [32]. Meanwhile, PE has a certain degree of volatility. During sample processing and determination, the moisture and PE evaporate rapidly, resulting in local volume shrinkage and changes in the vapor pressure, thus forming a porous structure. If the evaporation rate is uneven, it may further cause cracks or grooves. Furthermore, LIU et al. [30] demonstrated that the addition of essential oils might interfere with the cross-linking reactions (such as hydrogen bonds or covalent bonds) between polysaccharides and proteins, resulting in an uneven cross-linking network. Areas with low cross-linking density were prone to forming cracks or grooves under stress. In addition, the active components in PE (such as Perilla aldehyde, limonene, etc.) may interact dynamically with polysaccharides or proteins (such as hydrophobic aggregation and hydrogen bond recombination), forming discontinuous phases during the gel-curing process, thereby generating pores [32].

### 2.5. Results of X-Ray Diffraction (XRD)

**Figure 10:**
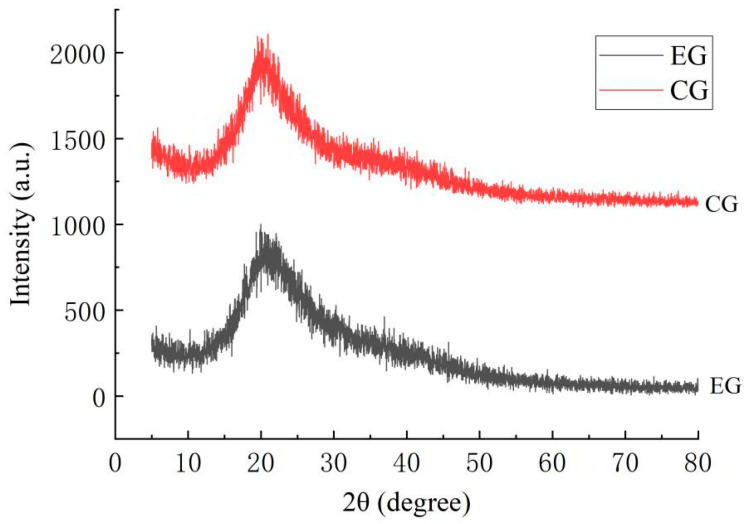
The XRD of composite edible gel films (CG: the control group without PE; EG: the experimental group with PE added).

Figure 10 shows the X-ray diffraction images of the EG (the experimental group with PE added) and CG (the control group without PE). The compatibility and the crystallinity of the composite gel films between the film-forming matrix and PE can be understood by X-ray diffraction. The EG did not show any new diffraction peaks, indicating that PE had good compatibility with other components. Both the CG and EG had a strong diffraction peak at 22.13°, indicating that the composite gel films had a certain degree of crystallinity and good compatibility among chitosan, gelatin, and GSE. The intensity of the diffraction peaks of the EG (sharp peaks at specific angles) was higher than that of CG, indicating that the crystallinity of the EG was relatively high and the amorphous structure had decreased. Generally speaking, the crystalline region formed a rigid structure through the orderly arrangement of molecular chains, which could effectively disperse external stress and thereby enhance TS. High crystallinity (CG) reduced the free volume within the material and prolonged the diffusion path of water vapor molecules. Meanwhile, the dense structure of the crystalline region hindered the penetration of water molecules, thereby significantly reducing the WVP. Furthermore, the O-H stretching vibration peak in the EG sample moved toward a higher wavenumber, indicating an enhanced hydrogen bond network. The synergistic effect of hydrogen bonds and high crystallinity further enhanced the rigidity and barrier properties of the material. The diffraction peak of the CG was relatively wide, indicating that its crystallinity was lower and the degree of disorder in the arrangement of molecular chains was higher. Generally speaking, an increase in the proportion of amorphous regions may lead to easier molecular chain sliding, resulting in a higher EAB and a lower TS. However, it is worth noting that PE may form nanoemulsions with chitosan, gelatin, or chitosan/gelatin mixed systems. Modifying materials through nanotechnology may simultaneously enhance the strength and toughness of the materials. While increasing the tensile strength, the elongation at break remained unchanged or even improved [17,18].

In conclusion, the high crystallinity and hydrogen bond network of the EG samples endowed them with excellent tensile strength and water vapor barrier properties. The EAB may vary accordingly, but it cannot achieve the maximum value simultaneously with TS. This is basically consistent with the single-factor results in Section 2.1 and the infrared spectroscopy analysis results in Section 2.3. At the same time, it once again confirms the necessity of establishing comprehensive indicators in this study to balance the indicators of TS, EAB, and WVP.

### 2.6. The Results of the Preservation Experiment

#### 2.6.1. The Change in Sensory Score During Storage

**Figure 11:**
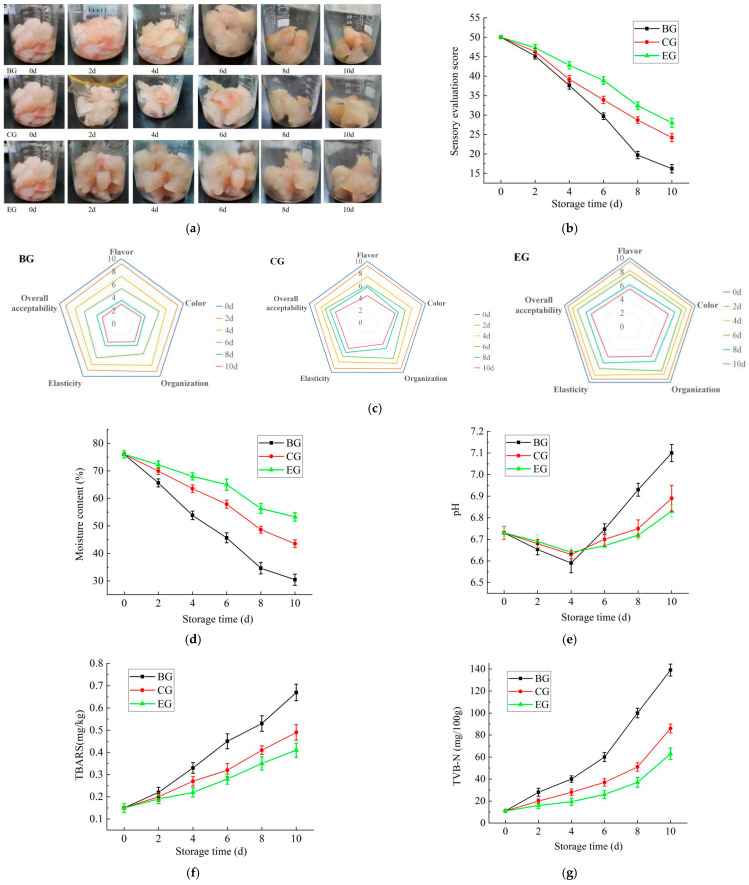
Effects of gel films on the physicochemical properties of grass carp during storage. (**a**) Effect of gel films on the shape of fish during storage. (**b**) Effect of gel films on the sensory evaluation score. (**c**) Radar map of sensory changes in fish during storage. (**d**) Effect of gel films on moisture content. (**e**) Effect of gel films on pH; (**f**) Effect of gel films on TBARS. (**g**) Effect of gel films on TVB-N; EG: the PE/GSE–gelatin/chitosan composite edible gel films with PE added at 10 μL/mL were used in the experimental group; CG: the GSE–gelatin/chitosan composite edible gel films without PE added were taken as the control group; BG: no preservation measures were used in the blank group (BG).

The gel films were utilized for the preservation of grass carp. The PE/GSE–gelatin/chitosan composite edible gel films with 10 μL/mL of PE added were used as the experimental group (EG), the GSE–gelatin/chitosan composite edible gel films without PE added were taken as the control group (CG), and no preservation measures were used in the blank group (BG). The changes in fish during storage are shown in Figure 11a, and the changes in sensory scores are shown in Figure 11b. As can be seen from the figure, the sensory scores of the EG, CG, and BG all show a downward trend with the extension of storage time (*p* < 0.05). Among them, the score of the BG decreased the fastest, the changes in color and tissue morphology were most obvious, followed by the CG, and the change in the EG was the smallest. This was because the gel films in the CG and EG played a good role in isolating oxygen and water, avoiding contact between the sample and the environment, reducing the loss of water in the fish, and effectively inhibiting the growth of bacteria. The addition of GSE played the role of an antioxidant and inhibited fish quality deterioration more effectively, which is consistent with the conclusions of Gao et al. [33]. The addition of PE in the EG further enhanced the oxygen and water barrier properties as well as the antioxidant and antibacterial activities of the gel films.

As can be seen from the radar chart, five indexes of flavor, color, organization, elasticity, and overall acceptability of all groups showed a significant downward trend during storage, and the downward trend of the BG was significantly greater than that of the CG and EG. The organization and elasticity of the BG showed a more significant decrease in the late storage period (4–6 d), while the organization and elasticity of the CG showed a more significant decrease after 6 d of storage. The indexes of the EG changed relatively little, and the changes were more uniform during 0–10 d of storage. The above results proved that the addition of PE had a positive effect on the sensory quality score of the gel films.

#### 2.6.2. The Change in Moisture Content During Storage

The changes in the moisture content of the fish samples after different preservation treatments during storage are shown in Figure 11d. The results showed that the initial moisture content of all samples was about 76%. The moisture content of all treated samples showed a decreasing trend during storage (*p* < 0.05), but the decreasing speed was different, especially in the late storage period (*p* < 0.05). The samples treated by the BG had the fastest water loss, followed by the CG, and the EG was the slowest. The decreasing rate of water content in the CG and EG in the early stage was slower than that in the late stage of storage, which was consistent with the results in 2.3.1. These results indicate that the composite gel films with PE can effectively reduce the loss of water in grass carp during storage. This may be because the active ingredients in PE (such as Perilla aldehyde, limonene, etc.) have good hydrophobicity and antioxidant properties, which can enhance the barrier properties of the gel films [19]. In addition, the interaction between PE, chitosan and gelatin can form a denser network structure, thereby reducing the evaporation of water [16]. In conclusion, the addition of PE can reduce the evaporation of water in grass carp during storage at 4 °C.

#### 2.6.3. The Change in pH Value During Storage

The pH value changes in samples under three different preservation treatments (BG, CG, EG) at different storage times (0 to 10 days) are shown in Figure 11e. As can be seen from the figure, the pH value of all the treated samples experienced a change of first decreasing and then increasing during storage. Among them, the pH value of the BG changed most significantly, and the final pH value was the highest. The CG and EG had similar pH trends, but the final pH of the CG was slightly higher than that of EG. This may be due to the decomposition of macromolecular proteins into alkaline compounds by microbial metabolism at the later stage of storage, resulting in an increase in pH [26]. Many studies have shown that GSE and PE have significant antioxidant and antibacterial activities, which can effectively inhibit the decomposition of nutrients and thus inhibit the excessive fluctuation in the pH value [34,35]. It can be seen that the CG and EG both showed good bacteriostasis, and the addition of PE can give the GSE–gelatin/chitosan composite edible gel films better bacteriostasis.

#### 2.6.4. The Change in Thiobarbituric Acid Reactive Substances (TBARS) During Storage

The TBARS is a measure of the degree of lipid oxidation and is commonly used to assess the freshness and oxidative stability of foods. Figure 11f shows the effects of different fresh-keeping treatments on the TBARS of samples. It can be seen from the figure that during 10 days of storage, the TBARS value of all treated samples increased with the extension of storage time. The lipid oxidation degree of BG-treated samples was the highest, the lipid oxidation degree of EG-treated samples was the lowest, and the increasing trend of TBARS was the most gentle. Studies have shown that phenolic components in PE have significant antioxidant properties, which can inhibit lipid oxidation, thus reducing the production of malondialdehyde (TBARS) and effectively maintaining the freshness of fish [32]. Similarly, Garcia et al. confirmed that the addition of lemongrass essential oil to chitosan/gelatin composite films could effectively inhibit the lipid oxidation of fish during storage at 4 °C for 10 days. Xiang et al. [36] proved that core–shell nanomembranes loaded with cinnamon essential oil could slow down the fat oxidation of refrigerated meat products. Teymoorian et al. [37] made use of chitosan/gelatin composite material to prepare a plastic wrap containing the essential oil of Dipterygium sinensis and successfully applied it to grape preservation. Zhang et al. [38] summarized that chitosan–essential oil composite film/coating showed superior antibacterial activity in food anticorrosive packaging and had broad application prospects.

#### 2.6.5. The Change in Total Volatile Base Nitrogen (TVB-N) During Storage

The TVB-N refers to the ammonia and amines produced by the decomposition of macromolecular substances such as proteins during the storage of animal foods due to the action of bacteria and enzymes. The content of volatile basic nitrogen can reflect the freshness of meat animal foods. As shown in Figure 11g, the TVB-N value of fish samples in the BG, CG, and EG changed significantly during storage (*p* < 0.05). With the extension of storage time, the TVB-N value of all treated samples increased significantly (*p* < 0.05), especially in the late storage period. As can be seen from the figure, the BG had the highest protein decomposition degree, and EG-treated samples had the lowest protein decomposition degree. It may be the active ingredients, such as phenols and aldehydes in PE, which can better inhibit the growth and reproduction of microorganisms and reduce the deterioration rate of proteins [16,19]. Therefore, during low temperature storage, PE/GSE–gelatin/chitosan composite edible gel films can achieve a good effect of grass carp preservation.

#### 2.6.6. Correlation Analysis of Indicator Changes

In order to further investigate the difference in the effects of different preservation treatments on the quality indexes of grass carp, correlation analysis was carried out on the physicochemical indexes of samples in the BG, CG, and EG during storage, and the results are shown in Table 4.

It can be seen from the data in Table 4 that there were certain differences in the correlation between the quality indexes in the BG, CG, and EG during storage, among which the difference in sample quality changes between the EG and BG was greater than that between the CG and BG. From a numerical point of view, the varying trends of water, pH, and TVB-N in the CG and EG were quite different, which indicated that the addition of PE had a great impact on the water, pH, and proteins of the samples. In other words, the addition of PE can effectively reduce the water loss of grass carp during storage, inhibit the fluctuation of pH, and inhibit the deterioration of proteins. This is consistent with the research results in Section 2.6.5.

## 3. Conclusions

In this study, PE/GSE–chitosan/gelatin composite edible gel films were successfully prepared, and the preparation process was optimized by a fuzzy mathematical method, single factor, and Box–Behnken response surface optimization combined with matlab analysis. The optimal conditions were that the addition of PE was 6.91 μL/mL, the addition of GSE was 0.45 mg/mL, and the gelatin was 1.63%. On this condition, the composite gels showed excellent mechanical properties (TS and EAB) and WVP. FTIR, SEM, and XRD analyses showed that molecular interaction occurred between PE and the matrix, and a small number of pores, could be observed in the cross-section. The preservation experiment proved that the composite gel films had a remarkable preservation effect on grass carp during storage at 4 °C. Compared with the BG and the CG, the EG showed better performance in sensory score, moisture content, pH value, TBARS value, and TVB-N value. Correlation analysis further confirmed the positive effect of PE/GSE–chitosan/gelatin composite edible gel films on the quality of grass carp. In summary, the composite gel films with PE added had good preservation performance and application potential, which provided a theoretical basis for the research and development of natural polysaccharide/protein composite edible gel films and promoted the development of green food-packaging materials.

## 4. Materials and Methods

### 4.1. Material

Perilla *frutescens* (L.) *Britt.* essential oil (PE), extracted from Perilla leaves (purity ≥ 98%, the main component was Perilla aldehyde, the content was 50–55%), was purchased from Jiangxi Supercritical Spice Oil Co., Ltd. (Yichun, China). The chitosan (N-degree of deacetylation ≥ 90%; the relative molecular mass was about 50–90 kDa) and gelatin, derived from fish scales (99% purity, chitosan (N-degree of deacetylation ≥ 90%, the relative molecular mass was about 50–90 kDa), relative molecular weight 60 KDa to 130 KDa), and the GSE (polyphenol content > 95%) were all food grade and purchased from Zhejiang Yinuo Biotechnology Co., Ltd., Lanxi, China. The glycerin, chloroform, carbinol, anhydrous ethanol, salicylic acid, boric acid, methylene blue, methyl red, 2-thiobarbituric acid, ferrous sulfate heptahydrate, etc., were analytically pure and brought from Xilong Scientific Co., Ltd. (Shantou, China). Water was purified with a Milli-Q water purification system (Millipore Co., Ltd., Burlington, MA, USA). Fresh Chinese Schemer (Hypophthalmichthys molitrix) (about 1.8 kg weight) was purchased from RT-Mart (Zhangzhou, China) and transported to the laboratory alive.

### 4.2. Preparation of PE/GSE–Chitosan/Gelatin Composite Edible Gel Films

A certain amount of chitosan was added to 50 mL of glacial acetic acid solution (2%). The mixed solution was stirred at 800 r/min for 5 min at 50 °C by the Magnetic stirrer (DF-101S, Shanghai Lichen Bangxi Instrument Technology Co., Ltd., Shanghai, China) to achieve a final concentration of chitosan solution at 1.0%. A certain amount of GSE was added into the chitosan solution and continued to be stirred for 5 min to make it completely mixed (solution 1). The gelatin solution was prepared by adding a certain amount of gelatin into 50 mL of deionized water at 50 °C and stirring it with 800 r/min for 20 min by Magnetic stirrer to make the mixture dissolve completely (solution 2). The above two solutions were mixed, and 1.75% of glycerol was added as a plasticizer. Then, a certain amount of PE was added into the mixing system, and the mixture was homogenized at high speed for 5 min at 10,000 r/min. The composite solution of PE/GSE–chitosan/gelatin was obtained. A certain volume of solution was evenly poured into the polytetrafluoron mold and dried in a blast drying oven at 50 °C for 7 h to obtain an edible gel film. The gel film was removed from under the mold for physical and chemical properties tests.

### 4.3. Process Optimization of the PE/GSE–Chitosan/Gelatin Composite Edible Gel Films

#### 4.3.1. Single-Factor Experiment

We investigated the effects of four single factors (the addition of GSE (0.1, 0.2, 0.3, 0.4, 0.5 mg/mL), the addition of PE (0, 5, 10, 15, 20 μL/mL), the addition of gelatin (1%, 1.25%, 1.5%, 1.75%, 2%), and the addition of glycerol (1.5%, 1.75%, 2%, 2.25%, 2.5%)) on the elongation at break (EAB), tensile strength (TS), and Water Vapor Permeability (WVP) of the composite edible gel films.

#### 4.3.2. The Box–Behnken Test and Matlab Analysis

According to the results of the single-factor analysis, the effect of the addition of glycerol on the TS and EAB of the composite gel films was small in the predetermined range. Three single factors, including the addition of PE, GSE, and gelatin, were selected for further response surface test design. The factor levels and the codes of the response surface test design are shown in Table 5.

The Matlab (MATrix LABoratory) software (R2021a), the optimization calculation method, and the graphics processing function of the algorithm language were used. Through the preparation of program M (program code), the four-dimensional interaction effects of the addition of PE, the addition of GSE, and the addition of gelatin, on the comprehensive score of the composite gel films, were calculated.

### 4.4. Characterization of PE/GSE–Chitosan/Gelatin Composite Edible Gel Films

#### 4.4.1. Thickness Test

The thickness of the gel films was tested with a digital micrometer (BMD-25D, Mitutoyo Mfg. Co., Ltd., Kawasaki, Japan). Five random regions were selected, the reading was accurate to 0.001 mm, and the average values were calculated.

#### 4.4.2. The Tests of TS and EAB

The texture analyzer determined the TS and EAB (CT3-10K, American Bollerfly Company, Middleboro, MA, USA) [26]. The gel films were cut into a dumbbell-shaped strip of 30 mm × 10 mm and were fixed with a fixture so that they were in a vertical state. We opened the tension test until the film was pulled off. The initial length of the film L0, the breaking length of the film L1, and the maximum tension F were recorded. Each sample was measured 3 times and averaged. The mechanical properties of the film were calculated according to Equations (2) and (3).

(2) $$\mathrm{TS}=\frac{F}{b\times d}$$

where

TS: tensile strength of the gel films, Mpa;

F: maximum tension of the gel films, N;

b: width of the gel films, mm;

d: thickness of the gel films, mm.

(3) $$EAB=\frac{L1-L0}{L0}\times 100$$

where

EAB—elongation at break of film, %;

L_1_—length of the film at fracture, mm;

L_0_—original length of film, mm.

#### 4.4.3. The Test of WVP

The test of WVP was made with reference to Xue et al. [26]. The gel films were sealed with a glass bottle containing the appropriate amount of deionized water. Then, the samples were placed at room temperature for 24 h before weighing. The WVP of the gel films was calculated according to Equation (4).

(4) $$WVP=\frac{∆m\times d}{A\times ∆t\times ∆p}$$

where

WVP—water vapor transmittance, 10^−5^ g cm/(KPa h kPa·cm^2^);

Δm—change in film mass, g;

d—film thickness, mm;

A—film area, m^2^;

Δt—change in time, d;

Δp—partial pressure difference in vapor inside and outside of the film, kPa.

#### 4.4.4. Comprehensive Evaluation of Fuzzy Mathematics

The membership function method in fuzzy mathematics was used to evaluate each evaluation index comprehensively and quantitatively. According to the different membership degrees of each evaluation index, a comprehensive index set was established. The membership degree values were calculated according to membership functions, and fuzzy comprehensive scores were further calculated. The membership degree (r_mn_) was ∈ [0, 1]. The larger the values of TS and EAB, the better, which were calculated according to Formula (5). The smaller the value of the WVP, the better, which was calculated according to Formula (6). The comprehensive score (Y) of the composite gel films was calculated according to Formula (7).

(5) $$r_{mn}=\frac{R-Rmin}{Rmax-Rmin}$$

(6) $$r_{mn}=\frac{Rmax-R}{Rmax-Rmin}$$

(7) $$Y=a1\times r_{1n}+a2\times r_{2n}+a3\times r_{3n}$$

where R was the index value; R_min_ and R_max_ were the minimum and maximum values of the same index, respectively. The a1~a3 and r_1n_~r_3n_ were the weights and membership degrees of TS, EAB, and WVP, respectively, and the values of a1~a3 were 0.2, 0.35, and 0.45, respectively.

#### 4.4.5. X-Ray Diffraction (XRD) Analysis

The gel films were cut to the appropriate size to ensure that their surface was flat and wrinkle-free. An X-ray diffractometer was used with a Cu target (producing Kα radiation with a wavelength of 0.1542 nm). Test conditions: tube voltage: 40 kV; tube current: 40 mA; scanning range: 2θ = 5–50°; scanning speed: 4°/min. By analyzing the position and intensity of the diffraction peaks, the crystallinity and phase structure of the gel films can be determined.

#### 4.4.6. FTIR Analysis

The gel films were cut to the appropriate size (usually a circle about 10–15 mm in diameter) to ensure that their surface was flat, free of wrinkles and impurities. We used a blank background as a reference. The gel films were placed on the sample holder and the infrared light passing through the gel films was measured. The test conditions were as follows: resolution: 4 cm^−1^; scanning times: 48; wavenumber range: 4000–400 cm^−1^.

#### 4.4.7. SEM Analysis

The SEM (Hitachi SU-8000, Tokyo, Japan) analysis was used to investigate the structural characterization of the composite gel films at an accelerating voltage of 20 kV. After drying, the film was cut to an appropriate size, and the film was fixed on the stainless-steel film with conductive adhesive, placed on the copper platform for gold spraying and testing, and the surface and cross-section structure were recorded [39].

### 4.5. Effect of PE/GSE–Chitosan/Gelatin Composite Edible Gel Films on Physicochemical Properties of Grass Carp During Storage

#### 4.5.1. Preservation Experiment

The fresh grass carp was humanely slaughtered with ice–water slurry (2.4 kg ice: 3.6 L water: 1.5 kg fish) for 20 min and immediately beheaded, scaled, eviscerated, and washed with tap water. The fish was collected and stored in ice within 24 h for further treatment. Before the experiment, the fish was cut into a 2 cm × 2 cm × 3 cm cube. The PE/GSE–gelatin/chitosan composite edible gel films with 10 μL/mL of PE added were used as the experimental group (EG), the GSE–gelatin/chitosan composite edible gel films without PE added were taken as the control group (CG), and no preservation measures were used in the blank group (BG). The above three groups of samples (BG, CG, EG) were stored in a constant temperature environment at 4 °C, and the sensory scores, moisture content, pH value, TBARS, and TVB-N of the samples were determined at each time period of 0, 2, 4, 6, 8 and 10 days, respectively.

#### 4.5.2. Measurement of Sensory Scores

Sensory scores were performed referring to the methods of Xue Shan et al. [24], and the criteria for sensory evaluation that were used are in Table 6. Five male and five female food students, who passed the training, were healthy, and had no bad habits, were selected to form a sensory evaluation group to conduct the sensory evaluation of odor, color, tissue, and elasticity on the fish samples (BG, CG, EG). Sensory scores were performed on the samples, according to Table 2. The results are expressed as averages.

#### 4.5.3. Measurement of Moisture Content

In total, 5 g of fish was weighed, denoted as m1, and dried in a drying oven at 105 °C. After 1 h, the fish was weighed, denoted as m2. The moisture content of the samples was calculated by Equation (8):

(8) $$\mathrm{Moisture}\;\mathrm{content}=\frac{m1-m2}{m1}\times 100\%$$

where

m1: the wet weight of samples, g;

m2: the weight of the samples after constant weight, g.

#### 4.5.4. Measurement of pH

The pH values of the samples were tested by a calibrated portable pH meter (Testo735-2, Testo AG, Schwarzwald, Germany). Each sample was measured at three different locations. The results are expressed as averages.

#### 4.5.5. Measurement of TBARS

The measurement of TBARS referred to Xue et al. [26]. First, 5 g of chopped fish was weighed, and the solution of 50 mL of 7.5% TCA and 0.1% EDTA was added. The mixture was heated in a water bath at 50 °C for 0.5 h. Then, we filtered the mixture with filter paper, obtained 3 mL of supernatant, added 3 mL of 0.02 mol/LTBA solution, and heated this in a boiling water bath for 45 min. After cooling, the above solution was centrifuged at 5000 rpm at 4 °C for 10 min; then, 3 mL of chloroform was added and swirled for 30 s. After layering, the absorbance of the supernatant was measured at 532 nm and 600 nm wavelengths. A mixture of 3 mL of TCA, 3 mL of TBA, and 3 mL of chloroform was used as the blank group. The TBARS value was the mass of malondialdehyde in the samples.

#### 4.5.6. Measurement of TVB-N

The determination of TVB-N referred to the method by Xue et al. [40]. TVB-N content was measured by distillation after the addition of MgO (0.2 g) to the samples (2.0 g) in a Kjeldahl distilling flask (Kjeltec 8400, FOSS, Hiller, Denmark, FOSSScino (Suzhou) Co., Ltd., Suzhou, China). TVB-N content was expressed as mg of nitrogen per 100 g of sample.

### 4.6. Statistical Analysis

The data collected were subjected to ANOVA using Excel^®^ 2010 software and Tukey tests (with a 95% confidence interval) to evaluate differences between the results. Matlab (MATrix LABoratory) software (R2021a) was used for interactive test data calculation and four-dimensional plotting. The Principal Component Analysis used SPSS 17.0. The optimization experiment was conducted by using design-expert 8.0.6.

## Figures and Tables

**Table 1:** The test and results of response surface optimization.

No.	A: PE Addition (μL/mL)	B: GSE Addition (mg/mL)	C: Gelatin Addition (%)	TS (MPa)	EAB (%)	WVP (10^−5^ g cm/(KPa h kPa·cm^2^))
1	−1 (5)	0 (0.4)	1 (2)	35.23	188.90	1.889
2	−1	−1 (0.3)	0 (1.75)	37.05	128.17	1.199
3	−1	0	−1 (1.5)	36.4	144.27	1.143
4	−1	1 (0.5)	0	46.02	147.13	1.284
5	0 (10)	1	1	22.36	193.47	2.018
6	0	1	−1	44.33	148.90	1.256
7	0	−1	1	37.05	162.97	1.702
8	0	−1	−1	21.97	127.03	1.113
9	0	0	0	25.09	168.27	1.179
10	0	0	0	24.44	170.73	1.179
11	0	0	0	26.26	162.70	1.179
12	0	0	0	23.79	172.53	1.179
13	0	0	0	24.57	166.07	1.179
14	1 (15)	1	0	39.26	141.27	1.817
15	1	−1	0	58.63	108.33	1.517
16	1	0	−1	15.73	140.27	1.529
17	1	0	1	63.18	182.27	2.454

**Table 2:** Membership degree values of fuzzy comprehensive evaluation indexes, and results of fuzzy comprehensive evaluation.

M	A	B	C	r_1n_	r_2n_	r_3n_	Y
1	−1	0	1	0.411	0.946	0.421	0.603
2	−1	−1	0	0.449	0.233	0.936	0.593
3	−1	0	−1	0.436	0.422	0.978	0.675
4	−1	1	0	0.638	0.456	0.872	0.68
5	0	1	1	0.140	1	0.325	0.524
6	0	1	−1	0.603	0.477	0.893	0.689
7	0	−1	1	0.449	0.642	0.561	0.567
8	0	−1	−1	0.136	0.22	1.000	0.553
9	0	0	0	0.197	0.704	0.951	0.712
10	0	0	0	0.184	0.733	0.951	0.721
11	0	0	0	0.222	0.639	0.951	0.696
12	0	0	0	0.170	0.754	0.951	0.726
13	0	0	0	0.186	0.678	0.951	0.702
14	1	1	0	0.496	0.387	0.475	0.448
15	1	−1	0	0.904	0	0.699	0.495
16	1	0	−1	0.000	0.375	0.699	0.442
17	1	0	1	1.000	0.868	0.000	0.504

Note: The r_1n_~r_3n_ represent the membership degrees of the TS, EAB, and WVP of the gel films, respectively. The Y represents the fuzzy mathematical comprehensive score calculated based on the weight and membership of TS, EAB, and WVP, according to Formula (7).

**Table 3:** Variance analysis of Y (fuzzy mathematical comprehensive score) regression equation.

Source	Sum of Squares	df	Mean Square	F Value	*p* Value	Significance
Model	0.1556	9	0.0173	34.90	< 0.0001	**
A	0.0548	1	0.0548	110.57	< 0.0001	**
B	0.0032	1	0.0032	6.54	0.0377	*
C	0.0022	1	0.0022	4.46	0.0725	
AB	0.0045	1	0.0045	9.06	0.0197	*
AC	0.0045	1	0.0045	9.06	0.0197	*
BC	0.0080	1	0.0080	16.17	0.0051	**
A^2^	0.0359	1	0.0359	72.44	< 0.0001	**
B^2^	0.0168	11	0.0168	33.81	0.0007	*
C^2^	0.0178	1	0.0178	35.99	0.0005	**
Residual	0.0035	7	0.0005			
Lack of fit	0.0028	3	0.0009	5.99	0.0582	
Pure error	0.0006	4	0.0002			
Cor total	0.1591	16				

Note: ** indicates that the difference is very significant, *p* < 0.01. * indicates a significant difference, *p* < 0.05.

**Table 4:** Correlation analysis of effects of different preservation treatments on quality indexes.

	Sensory Score	Moisture Content	pH	TBARS	TVB-N
BG	−0.9937	−0.9935	0.7995	0.9964	0.9716
CG	−0.9974	−0.9975	0.6503	0.9950	0.9472
EG	−0.9935	−0.9884	0.5027	0.9901	0.9250

**Table 5:** The factor levels and the codes of the response surface test design.

Factor	Levels		
	−1	0	1
The addition of PE (μL/mL)	5	10	15
The addition of GSE (mg/mL)	0.3	0.4	0.5
The addition of gelatin (%)	1.5	1.75	2
The addition of glycerol (%)	1	1.25	1.5

**Table 6:** Criteria for sensory evaluation.

Score	Odor	Color	Tissue	Elasticity
9–10	Fish smell was rich; no odor	White	Close section; clear texture	Rapid recovery after compression
7–8	Fish smell; no odor	Yellowish white	Partially close and clear	Slow recovery after pressing
5–6	Fish smell was light; slight odor	Yellowish	Not tight, but not loose	Recovery after compression is slow
3–4	Fishy and unpleasant smell	Grayish yellow	Soft section; partially loose	It is difficult to recover after pressing
1–2	Very fishy and smelly	gray	Oar-shaped and loose	It will not recover after compression

## Data Availability

The data presented in this study are openly available in the article.
